# A Text-Book of Medical Practice for Practitioners and Students

**Published:** 1905-09

**Authors:** 


**A Text-Book of Medical Practice for Practitioners and Students.** Edited by Wm. Bain, M.D. Pp. xxiv., 1011. London: Longmans, Green & Co. 1904.

This work is by several authors, and is on a novel plan, as short accounts of the anatomy, histology, and physiology of the systems or organs concerned are prefaced to the description of the various groups of diseases. At first sight this does not seem a very feasible scheme, as the amount of matter to be got into a text-book which is to cover the whole field of medicine is so great that to accomplish the task at all adequately within the limits of one volume is a difficult matter. We are bound to state, however, that the authors have achieved their task with a very great measure of success. There can be no question of the importance to the student of his bearing constantly in mind the anatomical and physiological knowledge he has painfully acquired when he is confronted with the problems of clinical medicine. By the time the student has become a practitioner experience will have impressed this upon him, but then he may find that his knowledge of the earlier subjects of the medical course has become rusty and needs refreshing. Both the student and the practitioner will find their needs in this respect met in the volume before us. The prefatory sections are not lengthy, contain all the essential facts, and are brought up to the level of the latest work in physiology. Being printed in smaller type than the strictly medical part of the text, much information is got into them; at the same time, this type is clear and there is no difficulty in reading it.

Of the medical sections we can also speak favourably, and have found the required information on many points for which we have consulted the book. Necessarily a treatise on medicine in one cannot compete in fulness with those in many volumes, and the need for condensation with the supply of the greatest possible amount of information involves a continuous statement of facts which demands close attention in perusal and occasionally makes the text difficult reading. The book strikes us as a very good practical guide to medicine; the descriptions of disease are terse, and the salient points in each are well brought out and impressed upon the reader's attention. The sections on pathology and diagnosis are sound, and the advice as to treatment good; in fact, some of the sections on treatment seem to us particularly good, especially that the course to be adopted is made clear and no space is wasted by ambiguous directions. The book is well printed in good type, and although the page is a large one the volume is not unduly bulky, and can be held with comfort. The illustrations are mostly confined to the anatomical and histological sections. The chapter on tropical diseases should be mentioned as an eminently useful one. In conclusion, we can confidently recommend the work as fulfilling well the purposes for which it is intended.

